# Outstanding Reviewers for *Nanoscale Advances* in 2019

**DOI:** 10.1039/d0na90021d

**Published:** 2020-04-24

## Abstract

We would like to take this opportunity to highlight the Outstanding Reviewers for *Nanoscale Advances* in 2019, as selected by the editorial team for their significant contribution to the journal.
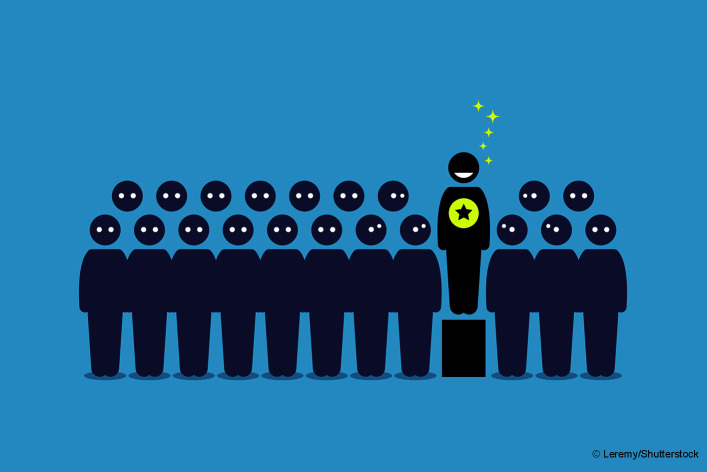

We would like to take this opportunity to thank all of *Nanoscale Advances’s* reviewers, and in particular highlight the Outstanding Reviewers for the journal in 2019, as selected by the editorial team for their significant contribution to *Nanoscale Advances*. We announce our Outstanding Reviewers annually and each receives a certificate to give recognition for their contribution. The reviewers have been chosen based on the number, timeliness and quality of the reports completed over the last 12 months.

 

**A message from Professor Dirk Guldi, Editor-in-Chief:**

Following recent years, *Nanoscale Advances* is, once again, recognizing the Outstanding Reviewers. Peer review is the essential tool to guarantee the quality and impact of *Nanoscale Advances*. Moreover, peer review depends on the excellence and timeliness of the reviews on top of the many demands that we face as active researchers. Carefully drafted reviews of work by our peers is at the heart of the peer review process as it provides a valuable service to the scientific community, in general, and to the readers of *Nanoscale Advances*, in particular. I want to add my thanks to these Outstanding Reviewers and also thank everyone who has reviewed manuscripts for *Nanoscale Advances*.

 

Dr Christopher Abram

Otto von Guericke University

ORCID: 0000-0003-3645-6977

 

Professor Katsuhiko Ariga

National Institute for Materials Science

ORCID: 0000-0002-2445-2955

 

Dr He Chen

Miami University

ORCID: 0000-0001-5426-769X

 

Professor Sanat Kumar

Columbia University

ORCID: 0000-0002-6690-2221

 

Dr Yupeng Li

University of Delaware

 

Dr Xuping Sun

University of Electronic Science and Technology of China

ORCID: 0000-0001-5034-1135

 

Dr Ke Xu

Hubei University of Arts and Science

 

Professor Yi-Jun Xu

Fuzhou University

ORCID: 0000-0002-2195-1695

 

Professor Han Zhang

Shenzhen University

ORCID: 0000-0002-0166-1973

 

Professor Junwei Zheng

Soochow University

ORCID: 0000-0002-6937-062X

 

We would also like to thank the *Nanoscale Advances* Editorial Board and Advisory Board and the nanoscience community for their continued support of the journal, as authors, reviewers and readers.

 

Dr Anna Rulka, Executive Editor

## Supplementary Material

